# Supplementary material for: Bi-level optimization of shared manufacturing service composition based on improved NSGA-II
Source: PLoS One. 2024 Jun 17;19(6):e0303968. doi: 10.1371/journal.pone.0303968 (PMC11182556; doi:10.1371/journal.pone.0303968)
Supplement: S1 File — (DOCX) [file pone.0303968.s001.docx]

**4.1 Determination of indicator weights - CRITIC method**

% CRITIC

clc;clear;

data=xlsread('E: \SR.xlsx');

data1=data;

index=[];

for i=1:length(index)

data1(:,index(i))=(max(data(:,index(i)))-data(:,index(i)))/(max(data(:,index(i)))-min(data(:,index(i))));

end

index_all=1:size(data1,2);

index_all(index)=[];

index=index_all;

for i=1:length(index)

data1(:,index(i))=(data(:,index(i))-min(data(:,index(i))))/(max(data(:,index(i)))-min(data(:,index(i))));

end

the=std(data1);

r=corr(data1);

f=sum(1-r);

c=the.*f;

w=c/sum(c);

| Model parameter | Value | Model parameter | Value |
| --- | --- | --- | --- |
| 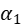 | 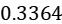 | 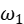 | 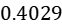 |
| 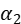 | 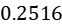 | 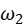 | 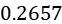 |
| 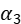 | 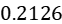 | 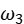 | 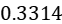 |
| 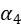 | 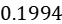 | 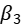 | 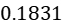 |
| 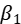 | 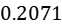 | 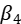 | 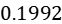 |
| 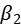 | 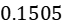 | 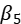 | 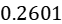 |

**5.1 Algorithm testing**

gd = generational_distance(y10, db3);

disp(['Generational Distance: ', num2str(gd)]);

%%

igd = calculateIGD(db3, y10);

disp(['calculateIGD: ', num2str(igd)]);

Table 2. GD and IGD values of the improved NSGA-II algorithm

| Iterations | Test the function | Improved GD | Improved IGD | Run time |
| --- | --- | --- | --- | --- |
| 100 | DTLZ1 | 0.038148 | 0.059894 | 13.175 |
|  | DTLZ2 | 1.091276 | 0.096658 |  |
|  | DTLZ3 | 0.594196 | 0.072488 |  |
| 300 | DTLZ1 | 0.486567 | 0.042839 | 38.242 |
|  | DTLZ2 | 0.42805 | 0.06704 |  |
|  | DTLZ3 | 0.505587 | 0.03498 |  |
| 500 | DTLZ1 | 0.507215 | 0.052317 | 63.836 |
|  | DTLZ2 | 0.509513 | 0.096307 |  |
|  | DTLZ3 | 0.487372 | 0.072732 |  |

Table 3. GD and IGD values of the traditional NSGA-II algorithm

| Iterations | Test the function | GD | IGD | Run time |
| --- | --- | --- | --- | --- |
| 100 | DTLZ1 | 2.58422 | 0.133574 | 44.262 |
|  | DTLZ2 | 2.44801 | 0.114911 |  |
|  | DTLZ3 | 2.58241 | 0.099055 |  |
| 300 | DTLZ1 | 2.43341 | 0.12879 | 135.591 |
|  | DTLZ2 | 2.55335 | 0.12061 |  |
|  | DTLZ3 | 2.60516 | 0.116259 |  |
| 500 | DTLZ1 | 2.46026 | 0.119588 | 212.699 |
|  | DTLZ2 | 2.51608 | 0.133373 |  |
|  | DTLZ3 | 2.47621 | 0.128891 |  |

**5.2 Sensitivity analysis**

figure;

plot(0.1:0.1:0.7, pc);

hold on;

plot(1:iteration, pc_old_history);


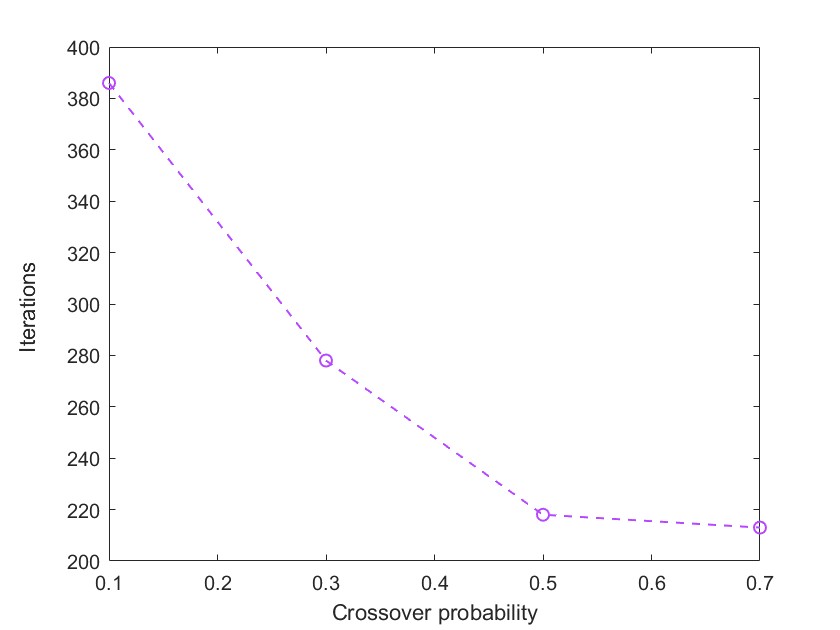


figure;

plot(0.01:0.01:0.04, pm);

hold on;

plot(1:iteration, pm_old_history);


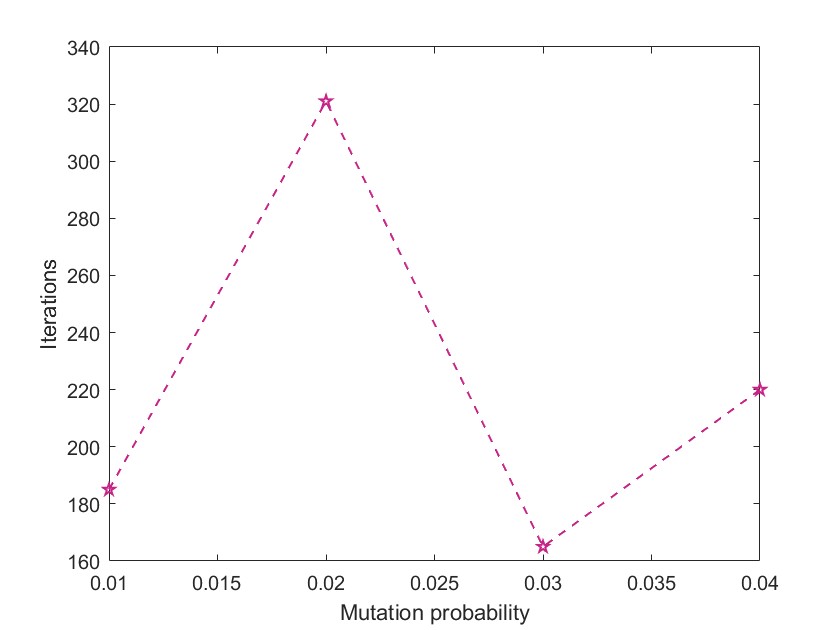


**5.4 Example Solution**

clear; close all; clear all

global NP iteration f_num x_num x_min x_max pc pm yita1 yita2 k bounds target

f_num = 3;

x_num = 5;

x_min = ones(1,x_num);

x_max = 4.*ones(1,x_num);

NP = 180;

iteration =200;

k = ceil(NP / 2);

yita1 = 7;

yita2 = 7;

pc = 0.7; pm = 0.03;

target=2;

parent_size = NP*0.6;

ud = 0.4; up = 1.6;

dimension=8;

chromo= initialize();

%%

[chromo]= object_fun2(chromo);

[F0, chromo] = non_domination_sort(chromo);

chromo = crowding_distance_sort(F0, chromo);

bounds=chromo;

for i = 1:iteration

chromo_parent = chromo(:,1:x_num+f_num+2);

parent_pop = select_parent(chromo_parent ,ud,up,parent_size);

child_pop= myga(parent_pop,bounds,i,iteration);

[child_pop]=object_fun3(child_pop);

chromo_co= combined_pop( chromo,child_pop);% chromo-parent_pop

[F_i, chromo_co] = non_domination_sort(chromo_co);

chromo_co = crowding_distance_sort(F_i, chromo_co);

chromo = elitism(chromo_co); %chromo = select_pop(chromo_co,NP);

end

%%

plot(chromo(:,x_num),chromo(:,x_num+f_num),'*')


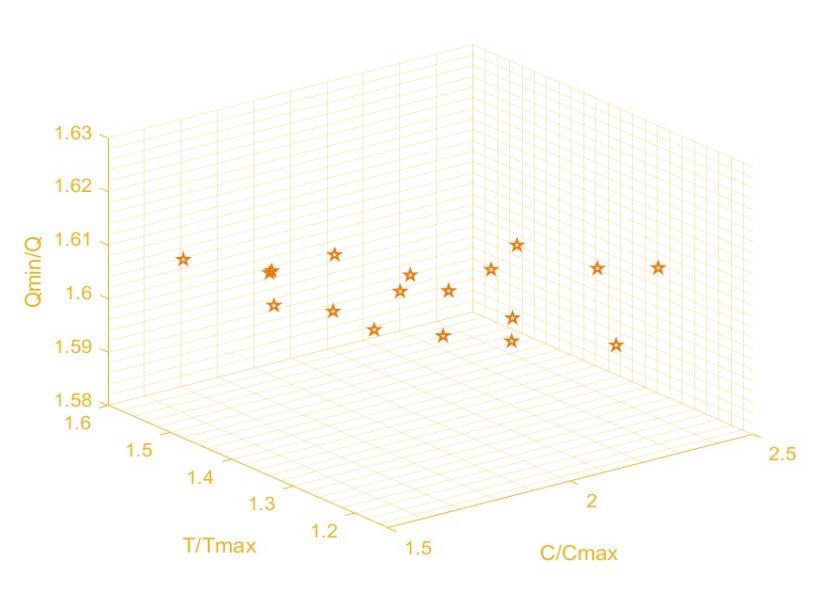


**5.5 Result analysis**

cc1;

oo1=chromo_sort(:,6)

iteration = 1:200;

plot(iteration, oo1,'g.- ',iteration,cc1,'b.-');

tt1;

chromo_1=sortrows(chromo_sort,7);

oo2=chromo_1(:,7);%time

iteration = 1:200;

plot(iteration, oo2,'g.- ',iteration,tt1, 'b.-');

qq1;

chromo_2=sortrows(chromo_sort,8);

oo3=chromo_2(:,8);%Q

iteration = 1:200;

plot(iteration, oo3,'g.- ',iteration,qq1, 'b.-');


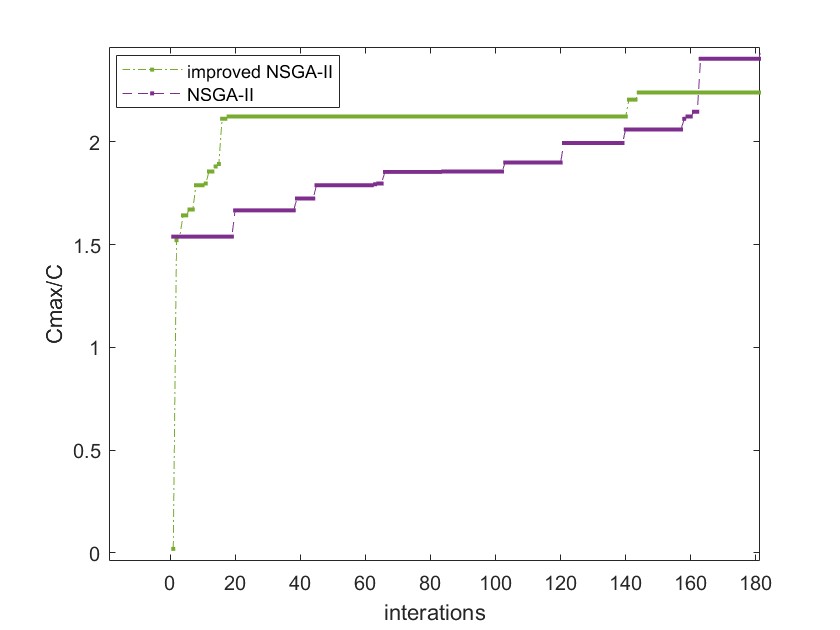


Figure 8. Comparison of cost convergence curves


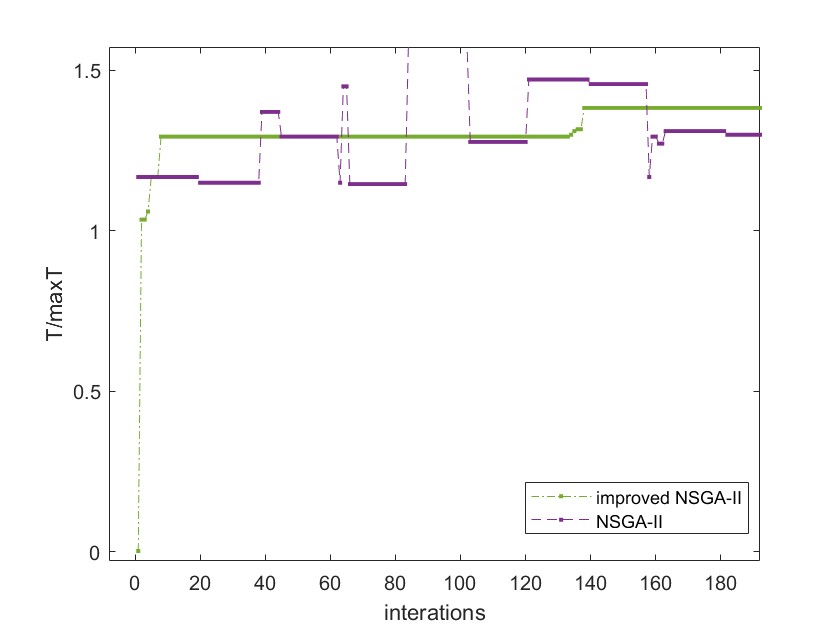


Figure 9. Comparison of time convergence curves


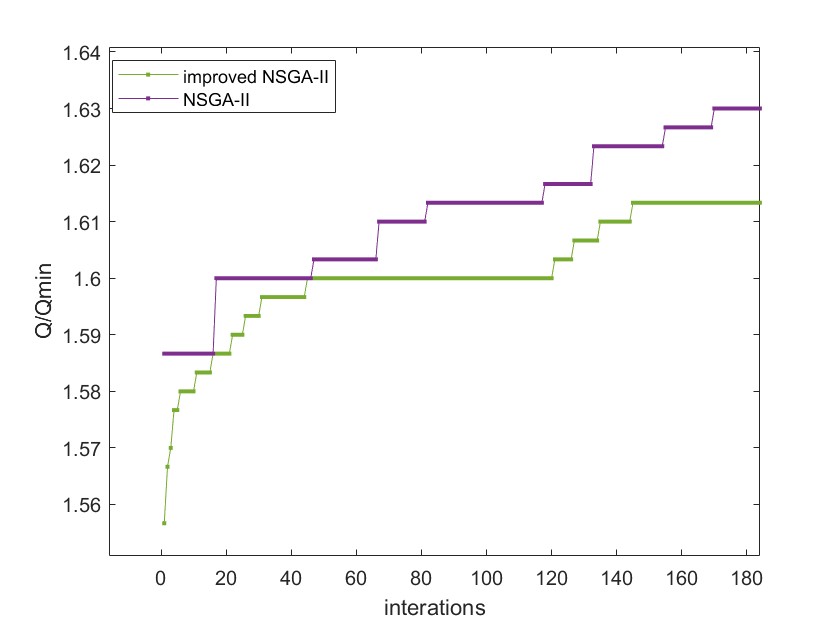


Figure 10. Comparison of convergence curves for quality compliance rate

function plotPareto(x)

switch x

case 1

zdt1 = readmatrix('improved nsga2.xlsx');

hold on

plot(zdt1(:,1),'r-')

hold on

plot(zdt1(:,2),'--')

legend('improved NSGA2 Pareto',' Pareto ')

case 2

zdt2 = readmatrix('iteration.xlsx');

hold on

plot(zdt2(:,1),'g--')

hold on

plot(zdt2(:,2),'b--')

hold on

plot(zdt2(:,3),'k--')

hold on

plot(zdt2(:,4),'m--')

legend('50th generation','100th generation','150thgeneration','200th generation')


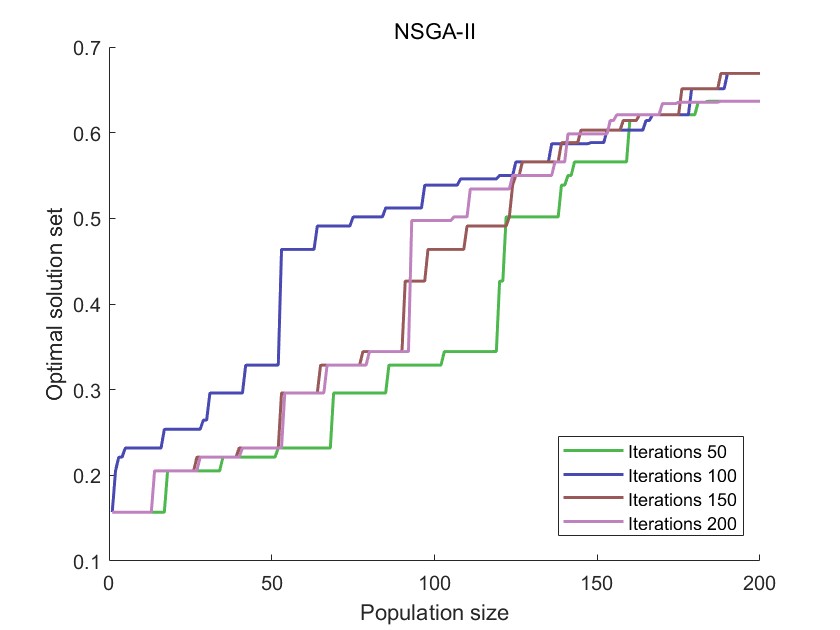


Figure 11. Using NSGA-II to obtain the optimal solution for iterations of 50, 100, 150, and 200


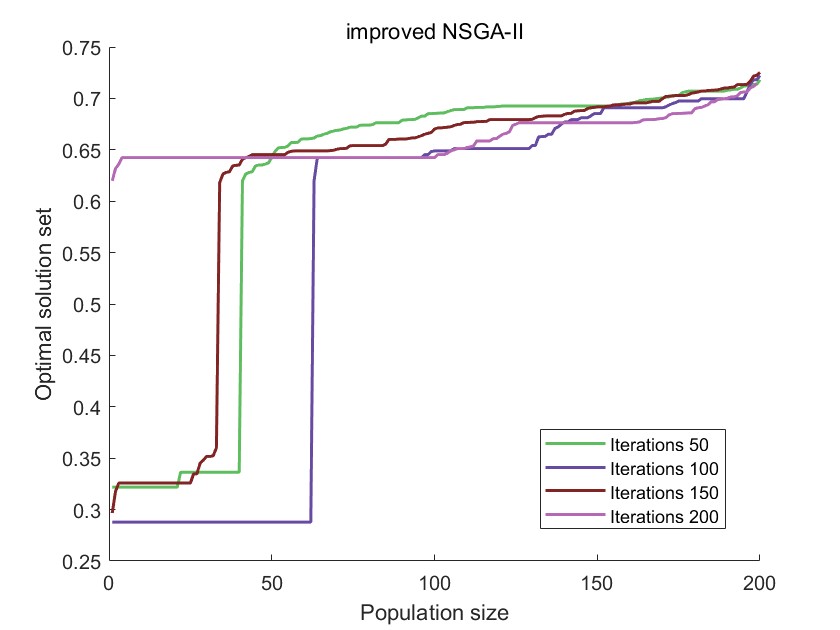


Figure 12. Using improved NSGA-II to obtain the optimal solution for iterations of 50, 100, 150, and 200
