# Supplementary material for: Bi-level optimization of shared manufacturing service composition based on improved NSGA-II
Source: PLoS One. 2024 Jun 17;19(6):e0303968. doi: 10.1371/journal.pone.0303968 (PMC11182556; doi:10.1371/journal.pone.0303968)
Supplement: Attachment — Submitted filename: Response to Reviewers 1-2 on PONE-D-23-41366.doc [file pone.0303968.s002.doc]

Dear Editor,

Thank you for allowing a resubmission of our manuscript (PONE-D-23-41366) for publication in *PLOS ONE*, with an opportunity to address the reviewers’ comments.

We are uploading (a) our point-by-point response to the comments, (b) an updated manuscript with red highlighting indicating changes, and (c) a clean updated manuscript without highlights.

We thank the referee and the editor very much for the comments and suggestions. We have made considerable effort on revising the paper based on these comments. For future communications, please contact either P. Liu at the addresses listed below.

Yours sincerely,

Peng Liu

Professor Peng Liu

School of Management

Shenyang University of Technology

Shenyang 110870

China

E-mail: liupeng@sut.edu.cn

**Response to the Comments of Reviewer #1 on PONE-D-23-41366**

We thank the referee very much for the comments and suggestions. They are very helpful for us to revise and improve the paper. The paper has been carefully revised according to the referee’s advice. We have made the following changes on the paper accordingly.

**Reviewer#1, Concern # 1:** As per the explanations presented in the manuscript, the ways to improve selection and mutation operators are clear; however, it is not clear how the crossover operator is modified such that the multi-objective upper-level optimization process is ameliorated. Please clarify.

**Author response:** As suggested by the referee, we have provided a detailed explanation in Section 3.2 of the revised paper on how the crossover operator has been improved.

We updated the manuscript with the yellow highlighted changes on page 14 in the revised paper.

**Reviewer#1, Concern # 2:** As the improved mutation strategy, is the probability of mutation decreased over the course of iterations or the mutation step? It is very important as the diversity of the population can be more toughly preserved during the optimization process if the mutation step is consistently decreased, not the mutation probability. Since the intensification ability of the algorithm has been strengthened in the proposed crossover strategy, the diversification strategy is logically expected to be ameliorated in the proposed mutation strategy. Please explain.

**Author response:** We thank the referee very much for the comment. In the improved mutation strategy, the mutation probability gradually decreases during the iteration process. The purpose of this strategy is to provide greater search space in the early stages of the algorithm, while gradually narrowing the search range in the later stages, thereby improving the search efficiency of the algorithm.

We updated the manuscript with the yellow highlighted changes on page 14 in the revised paper.

**Reviewer#1, Concern # 3:** In Table 5, isn’t the third column presented as “TC” instead of “CS”?

**Author response:** As suggested by the reviewers, we have modified the CS in Table 5.

We updated the manuscript with the yellow highlighted changes on page 18 in the revised paper.

**Reviewer#1, Concern # 4:** Why are the convergence curves depicted in the Figs. 6 to 8 ascending, while your goals in the multi-objective optimization of the upper-level functions are of minimization form? Furthermore, the label of the vertical axis in the Fig. 8 seems to be false. Please fix it.

**Author response:** Thanks so much for the careful inquiry and advice. We have modified the upper level functions and the coordinate axis labels in Figures 6 and 7.

We updated the manuscript with the yellow highlighted changes on page 21 in the revised paper.

**Reviewer#1, Concern # 5:**  How many decision variables are there in your multi-objective optimization problem?

**Author response:** We thank the referee very much for the comment. In multi-objective optimization problems, we include five decision variables corresponding to five shared manufacturing candidate tasks.

**Reviewer#1, Concern # 6:** It is recommended to first test the competence of your improved NSGA-II algorithm on a set of standard benchmark test functions and to compare the results with those of the conventional NSGA-II algorithm prior to go over solving the practical problem introduced in the manuscript.

**Author response:** We thank the referee very much for the comment. We have improved the three processes of parent selection, crossover and mutation of NSGA-II. The improved NSGA-II algorithm expands the search space when using parent selection based on linear ranking, improves the convergence speed when using guided crossover, and increases the fine-tuning ability of the algorithm when using non-uniform mutation. The improved NSGA-II is more efficient in seeking the optimal solution, converges to the global optimal solution faster and is more stable. Therefore, it is proved that the performance of the improved NSGA-II algorithm is better than that of the traditional one.

We updated the manuscript with the yellow highlighted changes on figure 9 and 10 in the revised paper.

**Reviewer#1, Concern # 7:**  The English of the manuscript can be and should be improved. There are considerable grammatical errors in the text of the manuscript that should be removed in the revised version.

**Author response:** We thank the referee very much for the comment. According to referee’s comments, we carefully checked and revised all grammar mistakes and typos.

**Reviewer#1, Concern # 8:**  Please include the final optimal solution (consisting of all the decision variables) obtained to solve the multi-objective upper-level optimization problem to a Table in the text of the manuscript.

**Author response:** Thanks so much for the careful inquiry and advice. We have liberated the optimal results obtained from all decision variables in Table 7, which shows the final result of removing duplicate data usefulness.

We updated the manuscript with the yellow highlighted changes on page 20 in the revised paper.

**Response to the Comments of Reviewer #2 on PONE-D-23-41366**

We thank the referee very much for the comments and suggestions. They are very helpful for us to revise and improve the paper. The paper has been carefully revised according to the referee’s advice. We have made the following changes on the paper accordingly.

**Reviewer#2, Concern # 1:** The composition flowchart in Figure 1 is not clear, and the authors should reorganize the figures to illustrate the critical factors in supply side and demand side.

**Author response:** Thanks so much for the careful inquiry and advice. We have made improvements to Figure 1 to make each section clearer, highlighting the key factors on the supply and demand sides.

We updated the manuscript with the yellow highlighted changes on page 5 in the revised paper.

**Reviewer#2, Concern # 2:** For the improved NSGA-II, the authors should consider the following issues, which are important to show the innovations of this paper.
(1) The detailed process of selection based on linear ranking, and it is necessary to report the reason of choosing this method.
(2) It is not reasonable for authors to evolve the individuals after cross, because the mentioned operations may reduce the diversity of populations, which is easy to fall into the local optimal solution.
(3) The authors should give the reason why Z Michalewicz’s non-uniform variation can be used to mutate the individuals.

**Author response:** We thank the referee very much for the comment. Our answers to the above three questions are as follows:

(1) The tournament selection method was originally used in the parent selection method. According to the tournament selection method, a predetermined number of people are chosen at random from the population to compete and obtain parents. The advantage is that anyone can enter the tournament, and a person with a high fitness level can appear as if they have numerous parents. This can assure fairness in parent selection, limit the search range of population evolution, and prevent local optima from forming. However, tournament selection has some limitations. The implementation of tournaments results in the selection of parents being comparable to random selection, which influences the speed of evolution throughout the population, and the search ability of the evolution process cannot be guaranteed. To ensure the algorithm's convergence speed, in the early stages of the algorithm, it is vital to assure its search capability, increase the search space, and avoid falling into local optima. In the subsequent stage, it is vital to raise the pressure on parent selection to ensure the algorithm's convergence so that the algorithm can better obtain the correct value. Select a parent selection method based on linear ranking.

Assuming N is the population size, linear selection ranking ranks individuals in descending order of fitness, followed by ascending order of crowding distance in NSGA-II. The lower the ranking (and the higher the fitness score), the greater the likelihood of selection. Then, using a relevant linear function, determine the selection probability for each individual.

(2) The traditional hybrid method is prone to becoming stuck in local optima, whereas guided crossover decreases this danger by introducing fresh mutations, allowing the algorithm to perform better in global searches. The proposed new crossover method-guided crossover, ensures that the evolutionary direction of offspring generated by each hybridization is determined based on the optimal direction between the two parent individuals and the two parent individuals' common genes, thereby increasing the algorithm's convergence speed.

(3) Non-uniform mutations can be sought across the full parameter space during the initial iteration step of genetic algorithms, increasing population variety and allowing the algorithm to identify a bigger solution space. As the number of iterations increases, non-uniform mutations do local searches around people, which contributes to the algorithm's evolutionary pace. This adaptive local search capacity can overcome the problem of uniform mutation, which makes it impossible to execute local searches in specific critical areas. Non-uniform mutation operators play an algebraic role, resulting in a wider range of mutations in the algorithm's early phases. The range of mutations shrinks as the number of evolutionary generations grows. This improves the algorithm's fine-tuning capacity, allowing it to more correctly locate the ideal answer.

We updated the manuscript with the yellow highlighted changes on page 14 in the revised paper.

**Reviewer#2, Concern # 3:** The authors should draw Figure 4 clearly, which should be corresponding to the detailed steps. Some Chinese symbols can be found in the flowchart.

**Author response:** We thank the reviewer for pointing out this issue. We have modified Figure 4 to correspond to the steps described above.

We updated the manuscript with the yellow highlighted changes on page 16 in the revised paper.

**Reviewer#2, Concern # 4:** The authors should introduce how to determine the parameters of NSGA-II because the proper combinations of parameters may have a great influence on the performance of algorithm.

**Author response:** We thank the referee very much for the comment. These parameters were referenced from earlier literature utilizing genetic algorithms and determined by continual debugging during the experiment.

We updated the manuscript with the yellow highlighted changes on page 19 in the revised paper.

**Reviewer#2, Concern # 5:** In this paper, there is only one numerical example, which is hard to verify the performance of the proposed method. Therefore, the authors should add more experiments to illustrated the advantages of the proposed method.

**Author response:** As suggested by the referee, we have compared the Pareto optimal solutions obtained with different iterations, as shown in Figures 9 and 10. By comparison, it can be seen that the improved NSGA-II performs better than traditional genetic algorithms, the iteration process is also relatively stable, and the selection of parameters is also appropriate.

We updated the manuscript with the yellow highlighted changes on page 23,24 in the revised paper.

**Reviewer#2, Concern # 6:** From Figures 6~9, it is hard to find that the improved NSGA-II is better than that of NSGA-II. Although the average of the improved method is better, the variance of the improved method is not good.

**Author response:** We thank the referee very much for the comment. We have added comparisons in Figures 9 and 10, and it can be seen that the improved NSGA-II is better than the NSGA-II.

We updated the manuscript with the yellow highlighted changes on page 23,24 in the revised paper.

**Reviewer#2, Concern # 7:** The authors should check the variables in this paper carefully, and some improper variables can be found easily, including the first variable in Equation 15, the last variable in Table 1.

**Author response:** We thank the referee very much for the comment. We carefully checked the variables in the text and made corrections.

We updated the manuscript with the yellow highlighted changes on page 13 in the revised paper.
