# Supplementary material for: Bi-level optimization of shared manufacturing service composition based on improved NSGA-II
Source: PLoS One. 2024 Jun 17;19(6):e0303968. doi: 10.1371/journal.pone.0303968 (PMC11182556; doi:10.1371/journal.pone.0303968)
Supplement: Attachment — Submitted filename: Response to Reviewers 1-3 on PONE-D-24-05998.doc [file pone.0303968.s003.doc]

Dear Editor,

Thank you for allowing a major revision of our manuscript (PONE-D-24-05598) for publication in *PLOS ONE*, with an opportunity to address the reviewers’ comments.

We are uploading (a) our point-by-point response to the comments, (b) an updated manuscript with yellow highlighting indicating changes, and (c) a clean updated manuscript without highlights.

We thank the referee and the editor very much for the comments and suggestions. We have made considerable effort on revising the paper based on these comments. For future communications, please contact either P. Liu at the addresses listed below.

Yours sincerely,

Peng Liu

Professor Peng Liu

School of Management

Shenyang University of Technology

Shenyang 110870

China

E-mail: liupeng@sut.edu.cn

**Response to the Comments of Reviewer #1 on PONE-D-24-05998**

We thank the referee very much for the comments and suggestions. They are very helpful for us to revise and improve the paper. The paper has been carefully revised according to the referee’s advice. We have made the following changes on the paper accordingly.

**Reviewer#1, Concern # 1:** The English should be improved. The whole proofreading is appreciated.

**Author response:** According to referee’s comments, we carefully checked and revised all text writing logic and spelling. The use of English in the revised paper is greatly improved.

We updated the manuscript with the yellow highlighted changes in the revised paper.

**Reviewer#1, Concern # 2:** The abstract should be re-written to enhance its quality. Adding the novelty and also numerical achievements in abstract is appreciated. Please put percentage improvement.

**Author response:** As suggested by the referee, we have improved the descriptions of the abstract. We have noted in the abstract the percentage improvement in operational efficiency and convergence speed of the improved NSGA-II algorithm compared to traditional algorithm.

We updated the manuscript with the yellow highlighted changes on page 1 in the revised paper.

**Reviewer#1, Concern # 3:** In abstract, you should mention the objective function is a cost-function which needs to be minimized.

**Author response:** We thank the referee very much for the comment. In the revised abstract, we have added the upper objective function as the function for minimizing and the lower objective function as the function for maximizing.

We updated the manuscript with the yellow highlighted changes on page 1 in the revised paper.

**Reviewer#1, Concern # 4:** What does CRITIC stand for? Please uses extracted from before the first usage of abbreviation. Please perform in all parts.

**Author response:** We thank the referee very much for the comment. The full name of the CRITIC is Criteria Importance Through Intercrieria Correlation. Before using abbreviations, we have indicated the full name of the CRITIC method.

We updated the manuscript with the yellow highlighted changes on page 1 in the revised paper.

**Reviewer#1, Concern # 5:**  I recommend the title needs changing because it would be more specified when you indicate your algorithm. It seems the current title does not convey the main goal especially NSGA-II.

**Author response:** We thank the referee very much for the comment. We have changed the title “Shared Manufacturing Service Composition Optimization Based on Bi-Level Programming” to “Bi-Level Optimization of Shared Manufacturing Service Composition Based on Improved NSGA-II”.

We updated the manuscript with the yellow highlighted changes on page 1 in the revised paper.

**Reviewer#1, Concern # 6:** The keywords are insufficient. Please add ample and the most relevant keywords.

**Author response:** According to referee’s comments, we have added “Improved NSGA-II algorithm” and “CRITIC method” to the keywords.

We updated the manuscript with the yellow highlighted changes on page 1 in the revised paper.

**Reviewer#1, Concern # 7:**  The used references are imperfect. Please rectify or update all references in terms of data, pages, doi, etc. In addition, more newly published and the most relevant papers should be added in some parts to increase the quality of paper and attracts more researchers’ attention on your manuscript. If possible for you, please omit old-fashioned or un-reputed papers and index-less journals from citation and reference list to enhance the manuscript authentication.

**Author response:** We thank the referee very much for the comment. We made changes to the references section and omit old-fashioned or un-reputed papers and index-less journals from citation and reference list to enhance the manuscript authentication.

We updated the manuscript with the yellow highlighted changes on pages 28-31 in the revised paper.

**Reviewer#1, Concern # 8:**  One of the most important thing that you used is NSGA-II algorithm. Please explain about it in literature review. Please cite this reference “K. Deb, A. Pratap, S. Agarwal and T. Meyarivan, "A fast and elitist multiobjective genetic algorithm: NSGA-II," in IEEE Transactions on Evolutionary Computation, vol. 6, no. 2, pp. 182-197, April 2002, doi: 10.1109/4235.996017.”

**Author response:** According to referee’s comments, we cite this reference “K. Deb, A. Pratap, S. Agarwal and T. Meyarivan, A fast and elitist multiobjective genetic algorithm: NSGA-II. IEEE Transactions on Evolutionary Computation, vol. 6, no. 2, pp. 182-197, April 2002, doi: 10.1109/4235.996017.” in Section 2.3.

We updated the manuscript with the yellow highlighted changes on page 4 and page 30 in the revised paper.

**Reviewer#1, Concern # 9:** In introduction section, please enlist contributions in bullet form, before paper structure is introduced.

**Author response:** According to referee’s comments, before introducing the structure of the paper, we added the main contributions made by this paper. The contributions of this paper are as follows: ①This paper establishes a bi-level programming model, which is different from previous bi-level programming models. The model targets the supply and demand sides of shared manufacturing, and the lower level indicators include service reliability, service credibility, and task coordination. The service reliability and credibility indicators are decomposed in detail, and the model represents more comprehensive interests. ②This paper uses the Criteria Importance Though Intercrieria Correlation (CRITIC) to determine the weights of indicators and make the upper and lower indicators more fair and objective. ③ This paper has enhanced the genetic algorithm’s selection, crossover, and mutation processes, as well as its efficiency and rate of convergence.

We updated the manuscript with the yellow highlighted changes on page 2 in the revised paper.

**Reviewer#1, Concern # 10:** The re-structure is necessary for your manuscript. It lacks literature review and related work. Please add related work.

**Author response:** According to referee’s comments, we have added literature review and related work in Section 2.

We updated the manuscript with the yellow highlighted changes on pages 3-4 in the revised paper.

**Reviewer#1, Concern # 11:** Related work should be extended. I recommend mention some papers below to improve the quality of current paper. Firstly, introduce NSGA-II, non-dominated solutions, and Pareto set concepts. Please use reference ““K. Deb, A. Pratap, S. Agarwal and T. Meyarivan, "A fast and elitist multiobjective genetic algorithm: NSGA-II," in IEEE Transactions on Evolutionary Computation, vol. 6, no. 2, pp. 182-197, April 2002, doi: 10.1109/4235.996017.”” with suitable citation.

The bi-level programming needs to be introduced with suitable reference. Please add couple of relevant papers in related work.

Service composition technology is used in several industries to reduce overall cost; also, this technology is engaged to improve reliability, security, etc. In IT industry, a decision model paper titled “An iterative mathematical decision model for cloud migration: A cost and security risk approach” was extended to indicate how service composite is beneficial for individuals and businesses.

Bi-objective web service composition algorithm entitled “Bi-objective web service composition problem in multi-cloud environment: a bi-objective time-varying particle swarm optimisation algorithm” was proposed to reduce overall cost and increase security in multi-cloud environment.
Recently published reliability –aware service composition method in paper titled “Reliability-aware web service composition with cost minimization perspective: a multi-objective particle swarm optimization model in multi-cloud scenarios” was proposed to increase system reliability.
A genetic algorithm was extended to solve web service composition problem in “Optimization of Automatic web services composition using genetic algorithm” for distributed systems.

**Author response:** According to referee’s comments, we have added references and citations to the literature on bi-level programming models in 2.1 and the concepts of NSGA-II, non-dominant ordering, and Pareto solution sets in section 2.3.

Service composition is the main topic of the current research concern. SHIRVANI M H el. [6] presented an iterative mathematical decision model for organizations to evaluate whether to invest in establishing information technology (IT) infrastructure on-premises or outsourcing IT services on a multicloud environment. In order to reduce overall costs and increase security in multi-cloud environment, SHIRVANI M H [7] formulated the web service composition problem as a bi-objective optimization problem with service cost and multi cloud risk perspectives in a ever-increasing multi cloud environment (MCE) in which each provider has its variable pricing strategy and different security level. TABALVANDANI M A N et al. [8] proposed a multi-objective particle swarm optimization model in multi-cloud scenarios to increase system reliability.

We updated the manuscript with the yellow highlighted changes on page 3 and page 28 in the revised paper.

**Reviewer#1, Concern # 12:** Is your algorithm heuristic or meta-heuristic? Why?

**Author response:** We thank the referee very much for the comment. The algorithm proposed in this paper is a meta-heuristic. The genetic algorithm itself is a meta-heuristic, which is inspired by the "survival of the fittest" in the process of biological evolution. By randomly generating an initial set of solutions, and then using genetic manipulation to cross and mutate, the quality of the solutions is continuously optimized until the optimal solution is reached. This algorithm is characterized by its versatility and flexibility, and can be applied to a variety of optimization problems. Although we have improved the process of selection, crossover, and mutation, and the improved NSGA-II algorithm has improved performance and efficiency compared with the traditional NSGA-II algorithm, it is still a meta-heuristic.

**Reviewer#1, Concern # 13:** Since you used GA, what is problem encoding? Termination criteria? Please put condensed sentences about my questions.

**Author response:** We thank the referee very much for the comment. It is used integer encoding to encode candidate manufacturing resource services to generate chromosome genes corresponding to individuals in the population. For example, for the candidate service composition, the corresponding chromosome encoding method is [1，3，1，2，3]. When the set maximum number of iterations is reached, the algorithm is terminated.

**Reviewer#1, Concern # 14:** Is your algorithm scalable?

**Author response:** We thank the referee very much for the comment. The improved NSGA-II algorithm proposed in this paper is scalable. The algorithm's performance growth is flat and able to remain within an acceptable range when dealing with larger or more complex problems. As shown in the table, as the number of iterations increases, the runtime of the algorithm increases at a flat rate.

| Iterations | Improved run time | Traditional run time |
| --- | --- | --- |
| 100 | 13.175 | 44.262 |
| 300 | 38.242 | 135.591 |
| 500 | 63.836 | 212.699 |

**Reviewer#1, Concern # 15:**  What is implementation environment? Please put condense explanation about implementation/simulation environment. More experiments are recommended to reach concrete results and conclusion.

**Author response:** We thank the referee very much for the comment. This article uses the improved NSGA-II algorithm to solve the model in the MATLAB 2022b computing environment (running on 12th Gen Intel (R) Core (TM) i5-12500H, 2.50 GHz, Windows 11 Home Chinese version, RAM16.0GB), with an initial population NP=200, maximum iteration interval=180, guided crossover probability pc=0.7, and non-uniform mutation probability pm=0.03. These parameters were referenced from earlier literature utilizing genetic algorithms and determined by continual debugging during the experiment.

We updated the manuscript with the yellow highlighted changes on page 22 in the revised paper.

**Reviewer#1, Concern # 16:**  The conclusion should be changed to “Conclusion and future direction”. Please put a condense sentence about limitation of your work. And what is the future direction?
In the current form, I cannot accept it publication, but it can be re-evaluated provided the issues are carefully addressed. Therefore, I recommend Major revision.

**Author response:** According to referee’s comments, we add limitation of our work and the future direction in the revised paper.

This paper mainly considers the research on the service combination optimization method from the perspective of the supply side and the demand side, but in the actual production and manufacturing process, the supply side of the shared manufacturing platform also has its own demand for the indicators of the whole production process. The next step will be to consider the interests of the three parties from the perspective of the shared manufacturing platform, improve the evaluation index system of the shared manufacturing service composition. For example, it is to incorporate the substitutability of services into the evaluation indicators required by the suppliers of shared manufacturing platforms. It is also to consider introducing a time decay function to handle service evaluation indicators.

We updated the manuscript with the yellow highlighted changes on page 27 in the revised paper.

**Response to the Comments of Reviewer #2 on PONE-D-24-05998**

We thank the referee very much for the comments and suggestions. They are very helpful for us to revise and improve the paper. The paper has been carefully revised according to the referee’s advice. We have made the following changes on the paper accordingly.

**Reviewer#2, Concern # 6:** It is recommended to first test the competence of your improved NSGA-II algorithm on a set of standard benchmark test functions and to compare the results with those of the conventional NSGA-II algorithm prior to go over solving the practical problem introduced in the manuscript.

**Author response:** As suggested by the referee, we select three test functions, DTLZ1, DTLZ2, and DTLZ3, and test the GD and IGD values of the traditional NSGA-II algorithm and improved NSGA-II algorithm for 100, 300, and 500 iterations, respectively. Test each function 10 times and take the average value, and record the algorithm running time for different iterations. The experimental results are shown in Tables 2-3.

Table 2. GD and IGD values of improved NSGA-II algorithm

| Iterations | Test the function | Improved GD | Improved IGD | Run time |
| --- | --- | --- | --- | --- |
| 100 | DTLZ1 | 0.038148 | 0.059894 | 13.175 |
|  | DTLZ2 | 1.091276 | 0.096658 |  |
|  | DTLZ3 | 0.594196 | 0.072488 |  |
| 300 | DTLZ1 | 0.486567 | 0.042839 | 38.242 |
|  | DTLZ2 | 0.42805 | 0.06704 |  |
|  | DTLZ3 | 0.505587 | 0.03498 |  |
| 500 | DTLZ1 | 0.507215 | 0.052317 | 63.836 |
|  | DTLZ2 | 0.509513 | 0.096307 |  |
|  | DTLZ3 | 0.487372 | 0.072732 |  |

Table 3. GD and IGD values of traditional NSGA-II algorithm

| Iterations | Test the function | GD | IGD | Run time |
| --- | --- | --- | --- | --- |
| 100 | DTLZ1 | 2.58422 | 0.133574 | 44.262 |
|  | DTLZ2 | 2.44801 | 0.114911 |  |
|  | DTLZ3 | 2.58241 | 0.099055 |  |
| 300 | DTLZ1 | 2.43341 | 0.12879 | 135.591 |
|  | DTLZ2 | 2.55335 | 0.12061 |  |
|  | DTLZ3 | 2.60516 | 0.116259 |  |
| 500 | DTLZ1 | 2.46026 | 0.119588 | 212.699 |
|  | DTLZ2 | 2.51608 | 0.133373 |  |
|  | DTLZ3 | 2.47621 | 0.128891 |  |

As the number of iterations increases, the convergence and diversity of the algorithm will be affected. According to Tables 2 and 3, it can be seen that the GD and IGD values of all test functions of the improved NSGA-II algorithm are smaller than those of the traditional NSGA-II algorithm. This indicates that the improved NSGA-II algorithm can approach the real optimal solution set faster during the iteration process, effectively utilize the search space, and obtain diverse solutions with faster convergence speed. The running time of the improved NSGA-II algorithm for solving is 69.99% less than that of traditional NSGA-II algorithm.

We updated the manuscript with the yellow highlighted changes on pages 16-18 in the revised paper.

**Reviewer#2, Concern # 7:** The English of the manuscript can be and should be improved. There are considerable grammatical errors in the text of the manuscript that should be removed in the revised version.

**Author response:** According to referee’s comments, we carefully checked and revised all text writing logic and spelling. The use of English in the revised paper is greatly improved.

We updated the manuscript with the yellow highlighted changes in the revised paper.

**Response to the Comments of Reviewer #3 on PONE-D-24-05998**

We thank the referee very much for the comments and suggestions. They are very helpful for us to revise and improve the paper. The paper has been carefully revised according to the referee’s advice. We have made the following changes on the paper accordingly.

**Reviewer#3, Concern # 1:** Clarity of Abstract:
The abstract is generally well-written, but it could be more concise. It would benefit from a tighter focus on the main contributions of the paper, such as the novel application of the CRITIC method and the improved NSGA-II algorithm.

**Author response:** We thank the referee very much for the comment. We have made modifications to the abstract and added applications to the two algorithms, as well as the efficiency and convergence speed by the improved NSGA-II algorithm.

We updated the manuscript with the yellow highlighted changes on page 1 in the revised paper.

**Reviewer#3, Concern # 2:** Methodology:
The authors should provide a more detailed explanation of how the CRITIC method is utilized to determine the weights of the indicators. For example, through the analysis of contrast and ambivalence, how to connect to the weighted formula.

**Author response:** As suggested by the referee, we have added a description of how to use the CRITIC method to obtain the weights. Since all values are as large as possible in the lower-level objective function of this article, there is no need to deal with negative indicators. All indicators need to be standardized and multiplied by the contrast intensity and conflict, and then the information-carrying capacity is calculated. Finally, the weights are calculated, and the larger the information-carrying capacity is, the greater the weight is.

We updated the manuscript with the yellow highlighted changes on page 22 in the revised paper.

**Reviewer#3, Concern # 3:** Case Study and Experimental Results:
It is essential to validate the proposed model with a comprehensive case study or multiple case studies. The experimental results should include a sensitivity analysis to demonstrate how the model responds to variations in input parameters.

**Author response:** As suggested by the referee, we have added sensitivity analysis in Section 5.2. In order to analyze the impacts of crossover and mutation probabilities on the solution results, while keeping other parameters constant, crossover probability = [0.1, 0.3, 0.5, 0.7] and mutation probability = [0.01, 0.02, 0.03, 0.04] were taken. The improved NSGA-II algorithm was run 10 times, and the required number of iterations to converge to the optimal solution was recorded and averaged. The result was shown in the Figures 5-6. From the graph, it can be seen that when the crossover probability is 0.7 and the mutation probability is 0.3, it can ensure that the improved NSGA-II converges to the optimal solution at the fastest speed.

Figure 5. The number of iterations required to converge to the optimal solution under different crossover probabilities

Figure 6. The number of iterations required to converge to the optimal solution under different crossover probabilities

We updated the manuscript with the yellow highlighted changes on pages 18-19 in the revised paper.

**Reviewer#3, Concern # 4:** Future Work:
While the discussion on future work is informative, it would be beneficial to include some direction on how the proposed model can be extended to accommodate dynamic changes in the shared manufacturing environment, such as fluctuating demand or the entry of new service providers.

**Author response:** We thank the referee very much for the comment. According to referee’s comments, we add the future work in the revised paper.

This paper mainly considers the research on the service combination optimization method from the perspective of the supply side and the demand side, but in the actual production and manufacturing process, the supply side of the shared manufacturing platform also has its own demand for the indicators of the whole production process. The next step will be to consider the interests of the three parties from the perspective of the shared manufacturing platform and improve the evaluation index system of the shared manufacturing service composition. For example, it is to incorporate the substitutability of services into the evaluation indicators required by the suppliers of shared manufacturing platforms. It is also to consider introducing a time decay function to handle service evaluation indicators.

We updated the manuscript with the yellow highlighted changes on page 27 in the revised paper.
